# Survey of physician experiences and perceptions about the diagnosis and treatment of fibromyalgia

**DOI:** 10.1186/1472-6963-12-356

**Published:** 2012-10-10

**Authors:** Serge Perrot, Ernest Choy, Danielle Petersel, Anna Ginovker, Erich Kramer

**Affiliations:** 1 Service de Médecine Interne et Centre de la Douleur, Hôtel-Dieu, Paris, France; 2 Section of Rheumatology, Cardiff University School of Medicine, Tenovus Building, Heath Park, Cardiff, Wales, UK; 3 Pfizer Inc, 235 East 42nd Street, New York, NY, 10017, USA; 4 Harris Interactive, Independence Way, Princeton, NJ, USA

## Abstract

**Background:**

Fibromyalgia (FM) is a condition characterized by widespread pain and is estimated to affect 0.5-5% of the general population. Historically, it has been classified as a rheumatologic disorder, but patients consult physicians from a variety of specialties in seeking diagnosis and ultimately treatment. Patients report considerable delay in receiving a diagnosis after initial presentation, suggesting diagnosis and management of FM might be a challenge to physicians.

**Methods:**

A questionnaire survey of 1622 physicians in six European countries, Mexico and South Korea was conducted. Specialties surveyed included primary care physicians (PCPs; n=809) and equal numbers of rheumatologists, neurologists, psychiatrists and pain specialists.

**Results:**

The sample included experienced doctors, with an expected clinical caseload for their specialty. Most (>80%) had seen a patient with FM in the last 2 years. Overall, 53% of physicians reported difficulty with diagnosing FM, 54% reported their training in FM was inadequate, and 32% considered themselves not knowledgeable about FM. Awareness of American College of Rheumatology classification criteria ranged from 32% for psychiatrists to 83% for rheumatologists. Sixty-four percent agreed patients found it difficult to communicate FM symptoms, and 79% said they needed to spend more time to identify FM. Thirty-eight percent were not confident in recognizing the symptoms of FM, and 48% were not confident in differentiating FM from conditions with similar symptoms. Thirty-seven percent were not confident developing an FM treatment plan, and 37% were not confident managing FM patients long-term. In general, rheumatologists reported least difficulties/greatest confidence, and PCPs and psychiatrists reported greatest difficulties/least confidence.

**Conclusions:**

Diagnosis and managing FM is challenging for physicians, especially PCPs and psychiatrists, but other specialties, including rheumatologists, also express difficulties. Improved training in FM and initiatives to improve patient-doctor communication are needed and may help the management of this condition.

## Background

Fibromyalgia (FM) is a disorder characterized by widespread pain [1]. The American College of Rheumatology (ACR) criteria classify patients as having FM if they have widespread pain (all four quadrants involved) for at least 3 months and at least 11 out of 18 tender points on digital palpation with a force of 4kg [2]. Although not essential for diagnosis, sleep disturbance, fatigue and morning stiffness are present in the vast majority of patients, and other quite common symptoms include cognitive problems, paresthesias, headache, irritable bowel or bladder and anxiety [2,3]. In fact, the ACR has recently published modified preliminary diagnostic criteria, following the 1990 classification criteria, which enable diagnosis without the tender point examination, and includes the severity of fatigue, waking unrefreshed and cognitive symptoms as core diagnostic assessments [4]. Fibromyalgia is estimated to have a worldwide prevalence in the order of 0.5-5% in the general population [5-8], and when classified using the ACR 1990 criteria is approximately seven times more prevalent in women than in men [5].

Fibromyalgia is generally considered to be the domain of the rheumatology specialty being a condition of muscular pain that for centuries was known as rheumatism, muscular rheumatism or non-articular rheumatism. The ACR issued classification criteria in 1990, and medical training in FM is generally encompassed within rheumatology training at an undergraduate level. Despite FM being a rheumatologic condition, patients very often consult with physicians from variety of specialties other than rheumatology [9]. In a survey of 800 FM patients in six European countries, Mexico and South Korea, patients most commonly presented to primary care physicians (PCPs), but also to neurologists and psychiatrists [9]. These same patients reported FM having trouble symptoms and a meaningful impact on their lives. In addition, it took an average of 2.3 years after experiencing symptoms and presentation to an average of 3.7 physicians before a diagnosis of FM was made [9]. This suggests that the diagnosis and management of FM might be a challenge to physicians. Indeed, while recommendations for FM diagnosis (the use of ACR criteria and the Fibromyalgia Impact Questionnaire) and treatment (aerobic exercise, cognitive behavioural therapy, amitriptyline, duloxetine, milnacipran, pregabalin) exist, these recommendations have yet to be adopted into a standard diagnostic or treatment algorithm [10]. This may be reflected in the significant differences that have been reported, in terms of FM diagnostic and treatment patterns, by physicians of different specialties in the United States [11].

Studies conducted in Canada have shown, through self-report, that physicians have differing attitudes towards the etiology, diagnosis, and treatment of FM [12,13]. Further, some physicians report difficulty in diagnosing/managing FM and feel they lack the knowledge and skill to manage FM patients [13]. Here, we report the findings of a large-scale international survey of physicians and their perceptions of FM. The underlying framework of this survey was that a general lack of physician awareness, recognition, and understanding of FM contributes to deficits in the diagnosis and management of FM patients. The goal of our survey was to assess the knowledge and understanding of FM among physicians of different specialties, in hopes of identifying specific elements which contribute to the difficulty in diagnosing and managing FM. Steps could then be taken to improve knowledge and understanding of these specific elements in hopes of improving patient care.

## Methods

### Approach

A survey was designed by Pfizer Inc in conjunction with Harris Interactive® to yield data on awareness, knowledge and perceptions regarding the diagnosis and treatment of FM among physicians of different specialties. Ethical approval for this study was not required as per the countries regulations.

### Sampling and recruitment

This survey was conducted in six European countries (France, Germany, Italy, The Netherlands, Spain, United Kingdom), Mexico and South Korea. To ensure data stability and enable robust analysis of PCPs versus specialist at a country level, it was planned to survey 100 PCPs and 100 specialists in each country. With a maximum potential sampling variance of 9.8 percentage points at the 95% confidence level, a sample size of 100 is generally accepted as providing reasonable data stability and statistical reliability. The sample of 100 specialists per country consists of roughly 25 interviews with each of the four specialties; rheumatologists, neurologists, psychiatrists and pain specialists. This specialist sample was designed to be analyzed at the aggregate level of “specialist” in each country, not by individual specialty.

Physicians were randomly sampled from proprietary databases, association lists, phone directories and other commercially available sample sources. Physicians were invited by phone or face-to-face to participate in the survey, explained the purpose of the survey and why they were chosen, and assured their responses would be kept confidential. To qualify for participation in the study, physicians must have had a primary specialty in general/family practice, internal medicine, rheumatology, neurology, psychiatry, anesthesiology (Mexico), pain management and/or sub-specialty in pain management or pain treatment; be in active patient practice and reside in one of the countries included in the study. Physicians who had not seen a FM within the last two years were included in the study, since the objective was to obtain a representative sample of country physicians and assess their general knowledge, understanding, and experience with fibromyalgia regardless of whether or not they are actively treating fibromyalgia patients. Physicians who completed the survey received a cash honorarium equivalent to 30-100$ US dollars, depending on physician specialty and country. In addition, multiple follow-up calls were made to encourage participation.

### Data collection

The survey ( Additional file 1)was a 15-min interview conducted via telephone, using computer-assisted telephone interviewing (CATI) technology in all countries except South Korea, where interviews were face-to-face in compliance with cultural norms. The interviews were conducted by trained interviewers using a fully structured questionnaire. The CATI system allows the programmed survey to be displayed on a computer screen so the interviewer can read each question, enter a response and then move on to the next screen. Skip patterns, depending on the answer to the previous question, are programmed into the survey and not reliant on the interviewer. The survey was conducted between February and April 2008. At the time there were no medications approved for FM in any of the countries surveyed. The questionnaire included questions about physicians’ medical practice, their behaviors and perceptions related to diagnosis and treatment of FM. Questions were generally answered as yes/no or on 5-point Likert scales, e.g., 1 = strongly agree, 2 = somewhat agree, 3 = neither agree nor disagree, 4 = somewhat disagree, 5 = strongly disagree. Questions included lists of possible answers that were generally read and selected by the physicians. Not all physicians received the same identical set of questions, as some were contingent on whether the physician had seen patients with FM in the last 2 years. Specific details of questions and responses are provided in the results below.

### Data analysis

Data from all countries were pooled for presentation in this publication. Statistical significance at the 95% confidence level was tested using Student’s *t* test on proportions and means. The survey was developed by Harris Interactive in close cooperation/partnership with Pfizer Inc and the European Network of Fibromyalgia Associations (ENFA).

## Results

### Physician participation

In total, 1622 physicians were interviewed. In each country the target number of physicians in each specialty was recruited and interviewed, and in some countries slightly more were interviewed (up to 103 PCPs and up to 27 in a specific specialty). Characteristics of the sample according to specialty are shown in Table 1.

**Table 1:** Characteristics of the physician sample

	**PCP (n=809)**^**a**^	**Rheumatologist (n=206)**^**b**^	**Neurologist (n=201)**^**c**^	**Psychiatrist (n=204)**^**d**^	**Pain specialist (n=202)**^**e**^
**Years in practice,** mean n	20.1^c^	18.9	17.8	20.8^c^	19.5
**Total patients per month estimate,** mean n	614.6^b, c, d, e^	355.4^d, e^	322.3^d^	215.2	267.1^d^
**Seen FM patient in the last 2 years**, yes %	79^d^	93^a, c, d, e^	79^d^	63	87^a, c, d^
FM patients in the last 2 years*,*mean n*	31.5	126.9^a, c, d^	53.1^a, d^	26.0	86.8^a, d^
FM patients in the past 2 years who are women*,*mean %*	83.6	87.7^a, c, d, e^	84.1	83.9	82.3
**Find FM difficult to diagnose**^**†**^**,** %	61^b, c, d, e^	31	50^b^	53^b^	50^b^
**Training in FM**^**‡**^					
*Mean score*	2.2	2.7^a, c, d, e^	2.5^a, d^	2.1	2.5^a, d^
*Excellent, %*	4	16^a, c, d^	9^a, d^	3	10^a, d^
*Adequate, %*	33	52%^a, d^	45%^a, d^	32%	46%^a, d^
*Very little, %*	45%^b, c, d, e^	23	33^b^	37^b^	29
*None at all, %*	17^b^	9	13	27^a, b, c, e^	14
**Knowledge about FM**^**§**^					
*Mean score*	2.7^d^	3.2^a, c, d, e^	2.8^a, d^	2.6	2.9^a, d^
*Very knowledgeable, %*	8	33^a, c, d, e^	15^a^	9	17^a, d^
*Knowledgeable, %*	55^d^	54^d^	54	45	59^d^
*Not very knowledgeable, %*	34^b, c, e^	12	25^b^	39^b, c, e^	20^b^
*Not at all knowledgeable, %*	2	1	5^a, b^	7^a, b, e^	2

*Among those physicians who have seen patients with FM in the last 2 years.

^†^Somewhat or very difficult to diagnose.

^‡^Self-rating of training in FM: 1 = none at all; 2 = very little; 3 = adequate; 4 = excellent.

^§^Self-rating of knowledge of FM: 1 = not at all knowledgeable; 2 = not very knowledgeable; 3 = knowledgeable; 4 = very knowledgeable

^a,^^b,^^c,^^d,^ and ^e^ indicate statistically significant difference (*P*<0.05) among the subgroups (i.e., physician specialties) being analyzed.

### Physician practice characteristics

Overall, the average duration of clinical practice was approximately 20 years, and across specialties the means were generally similar, although the difference between neurologists and each of the PCPs and psychiatrists was statistically significant. Primary care physicians saw the greatest number of patients per month (mean, 615), significantly exceeding all other specialties. Among the other specialties rheumatologists and neurologists saw on average >300 patients/month, while psychiatrists and pain specialists saw >200/month. Almost all of the rheumatologists had seen a patient with FM in the last 2 years, and the frequency was significantly greater than all other specialties except the pain specialists, most of whom had also seen a patient with FM. In the self-rating of training in FM among group differences were noted, with PCPs and psychiatrists indicating poorer levels of training than the other specialties, and rheumatologists generally reporting significantly better training than others. Self-reported knowledge of FM was likewise variable across specialties, with rheumatologists having the highest ratings and PCPs and psychiatrists having the lowest.

### Physician attitudes towards diagnosing fibromyalgia

**Figure 1:**
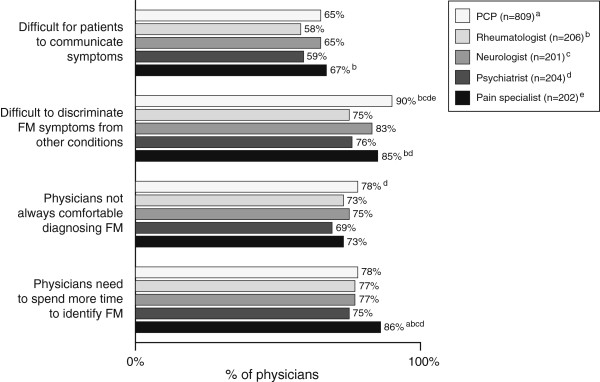
Physicians agreeing* with the reasons proposed as contributing to difficulty in diagnosis. *Those who strongly or somewhat agreed. ^a,^^b,^^c,^^d,^ and ^e,^ indicate statistically significant difference (*P*<0.05) among the subgroups (i.e., physician specialties) being analyzed.

A notable percentage of physicians (53%) responded that they found FM somewhat or very difficult to diagnose (Table 1). The percentages of physicians by specialty who agree with the reasons proposed as contributing to difficulty in diagnosis are shown in Figure 1. Overall, more than half (64%) the physicians across specialties considered that it was difficult for patients to communicate their FM symptoms. Most (85%) physicians found FM symptoms difficult to discriminate from other conditions, and 75% were not always comfortable with diagnosing FM. Compared with all other specialties, PCPs were significantly more likely than others to find it difficult to discriminate FM symptoms from other conditions; pain specialists were significantly more likely to feel physicians needed to spend more time to identify FM. Among those physicians who have seen FM patients in the past 2 years, the overall patterns of responses to the questions on diagnosis were similar for the entire sample, although differences across specialties were not as great (data not shown).

**Figure 2:**
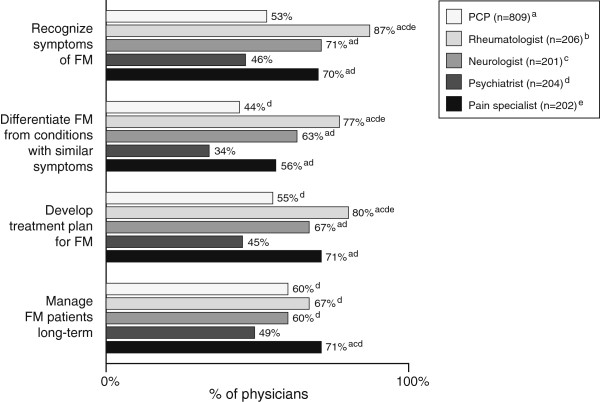
Physicians who reported being confident* in aspects of FM diagnosis and management. *Those who were very confident or confident. ^a^, ^b^, ^c^, ^d^, and ^e^ indicate statistically significant difference (*P*<0.05) among the subgroups (i.e., physician specialties) being analyzed.

The percentages of physicians who reported confidence in recognizing the symptoms of FM and in differentiating FM from conditions with similar symptoms was variable across specialities (Figure 2). Significantly more rheumatologists were more confident than the other specialities in both recognition of symptoms and differentiation from other conditions.

In total, 48% of all respondents were aware of the ACR Fibromyalgia Classification Criteria [2]. These criteria classify a patient as having FM if they have widespread pain (all four quadrants involved) for at least 3 months and at least 11 out of 18 tender points on digital palpation with a force of 4kg. Awareness of the ACR criteria was highest among rheumatologists (83%), which was significantly greater than all of the other specialties. Awareness among other specialties was as follows: pain specialists (56%), neurologists (55%), PCPs (40%) and psychiatrists (32%). Of the 725 physicians who were aware of ACR criteria and had treated an FM patient in the last 2 years, 71% said they use the ACR criteria to identify FM in their clinical practice. Use of ACR criteria was highest among rheumatologists (83%), followed by pain specialists (77%), PCPs (69%), neurologists (65%) and psychiatrists (41%).

### Physician attitudes towards treating fibromyalgia

Physicians who had seen FM patients in the past 2 years identified symptoms from a list of 14 that they felt were not adequately treated by currently available treatment options (Table 2). Generally, the pattern of responses was similar across specialties, with fatigue, chronic pain and concentration difficulties being the three most commonly selected in each group.

**Table 2:** Percentage of physicians who believe that available treatments at the time did not adequately treat common specific symptoms of FM*

	**PCP (n=639)**^a^**, %**	**Rheumatologist (n=192)**^b^**, %**	**Neurologist (n=158)**^c^**, %**	**Psychiatrist (n=129)**^d^**, %**	**Pain specialist (n=176)**^e^**, %**
**Fatigue**	51	54	49	50	51
**Chronic widespread pain**	50	60^a^	51	56	57
**Difficulty concentrating**	44	47	44	40	47
**Numbness/tingling**	40	38	35	43	40
**Feelings of depression**	37	38	40	33	42
**Stiffness**	35	33	37	37	39
**Problems sleeping**	31	28	34	23	36^d^
**Feelings of anxiety**	31	34	27	25	29
**Headaches**	28	30	31	22	25

*At the time the survey was conducted no medications for FM were licensed in any of the countries surveyed.

^a^, ^b^, ^c^, ^d^, and ^e^ indicate statistically significant difference (*P*<0.05) among the subgroups (i.e., physician specialties) being analyzed.

Significantly more rheumatologists than the other four specialties were confident in developing a treatment plan for FM (Figure 2), with approximately half the PCPs and psychiatrists expressing confidence in doing so. Differences among specialties in the percentages expressing confidence in managing FM patients long-term were not as great, mainly because fewer rheumatologists expressed confidence in managing FM patients long-term.

## Discussion

This survey collected data using structured questionnaires from 1622 physicians across five specialties from eight countries. Based on the average time in practice (~20 years), the sample can be considered to be experienced in the practice of medicine. Expectedly, PCPs saw far more patients/month than the other specialties, with frequency of patient consultations that approximates to 25 to 30 patients per working day. Psychiatrists and pain specialists saw fewer than half this number of patients, with rheumatologists and neurologists seeing just over half. This is consistent with what are accepted “norms” in medical practices, whereby PCPs see many more patients than other specialties in a typical working day.

As FM is classified as a rheumatologic condition (ACR) [2,4] and it is internationally recognized as a discrete condition in its own right (WHO ICD-10 code M79.7), it is not surprising that almost all rheumatologists (93%) surveyed reported seeing a patient with FM in the past 2 years as did most pain specialists (87%). Although the reported frequencies were lower among the other specialties, over two-thirds of these physicians reported seeing a patient with FM at some time in the last 2 years. Given that the prevalence of FM is estimated at 0.5-5% of the general population [5-8], that patients with FM are known to be frequent presenters to medical practitioners and high utilizers of health care resources [14] and that patients report difficulty in receiving a diagnosis and see multiple physicians [9], it is not surprising that the majority of neurologists and psychiatrists had also seen patients with FM in the past 2 years.

The finding that 21% of PCPs reported they had not seen a patient with FM in the past 2 years was a little surprising, as a PCP might be expected to have 1500–3000 patients in the community in their care [15] and that it has been estimated that FM constitutes 5% of a PCP’s consultation rate [16,17]. On this basis one would expect almost all PCPs to see a patient with FM in a 2-year period. The difficulties in making a diagnosis reflected by the PCP responses may explain why almost one in five of the PCPs claimed not to have seen a patient with FM in the last 2 years. Among those physicians who reported seeing a patient with FM in the last 2 years, the number of patients they saw, as expected, was quite variable across specialties, with rheumatologists seeing the highest number (average, 127), followed by pain specialists (average, 87), with psychiatrists seeing the fewest patients (average, 26 in 2 years).

Over half (53%) of the physicians said that they had difficulty diagnosing FM. Primary care physicians were significantly more likely than all other specialists to report that it is somewhat or very difficult to diagnose FM; rheumatologists were the least likely to report this. This is not surprising, given that a notable proportion (54% overall) said they received inadequate training in FM, and many (32% overall) still considered themselves not to be very knowledgeable about FM despite their experience in medical practice. Expectedly, the rheumatologists had the highest ratings on their level of training and knowledge. The finding that <60% of specialists (other than rheumatologists) were aware of the ACR criteria for FM classification that describes the tender point examination is consistent with the expressed inadequacy in training. It is worth noting that the modified preliminary diagnostic ACR criteria published in 2010, does not include a mandatory tender point examination to reach a diagnosis [4]. This may make the condition easier for clinicians to diagnose in the future if they receive training in using the revised criteria. The PCPs and psychiatrists reported the lowest ratings for training and knowledge, which probably reflects the fact that there are many more prevalent conditions that these specialties have to manage and have higher priority in their training. Nonetheless, the self-reported inadequacy of training and paucity of knowledge reported by many PCPs, in particular, is concerning. In many health care systems, including some of those surveyed, PCPs are the gatekeepers who provide referrals to other specialties and are very often the ongoing providers of care for patients with FM. Thus, PCPs having adequate training and good knowledge of FM is desirable.

In addition to training and knowledge of FM, the physicians were asked to rate factors that contributed to making FM diagnosis difficult. Over half (64%) the physicians overall agreed that it was difficult for patients to communicate their symptoms. This concurs with findings from the companion survey in which 59% of patients with FM said they found it difficult to communicate their FM to physicians [9]. The communication difficulties between patients and their doctors may, in part, be due to the nature of the disorder itself, as its presentation is variable across patients with a variety of symptoms, in addition to characteristic and otherwise unexplained chronic widespread pain, fatigue and sleep disturbance being manifest [18]. The fact that >75% of each of the specialties agreed that the need to spend more time to identify FM contributed to difficulty with diagnosis is consistent with the companion survey in which 74% of patients said that physicians need to spend more time to diagnose FM, which may be a factor that impacts the communication between patients and physicians.

Discomfort with making a FM diagnosis and difficulty discriminating FM symptoms from other conditions contributed to diagnostic difficulties according to most physicians. Notably, almost all PCPs (90%) reported that difficulty in discriminating FM symptoms from other conditions contributed to diagnostic difficulties, and the frequency was significantly greater than the other specialties. Consistent with this, only 44% of PCPs and only 34% of psychiatrists reported being confident in differentiating FM from conditions with similar symptoms. By contrast most rheumatologists were confident in recognizing FM symptoms (87%) and discriminating these from conditions with similar symptoms (77%), an expected finding, given the level of training and exposure to FM patients they will have likely received. The percentages of neurologists and pain specialists who reported being confident in recognition and discrimination of FM symptoms fell between the levels reported by the rheumatologists and the PCPs/psychiatrists, probably reflecting greater level of expertise in managing chronic painful conditions. Overall, these findings highlight the need for improved physician training in the diagnosis of FM, particularly PCPs and psychiatrists.

Least confident in the development of a treatment plan and in the management FM patients long-term were the psychiatrists, with fewer than half the respondents expressing confidence in either aspect of management. The levels of confidence were a little higher among the PCPs, but a significant minority did not express confidence. While most rheumatologists (80%) were confident in developing a treatment plan, only two-thirds were confident they could manage FM patients long-term. The fact that approximately half of each of the specialty groups surveyed believed that current treatments did not adequately treat cardinal symptoms of FM, such as chronic widespread pain and fatigue, offers some explanation that a notable proportion of physicians did not express confidence in managing FM long-term.

Overall, our findings are similar to those reported by Hayes et. al. [13], who examined the attitudes and experiences of Canadian physicians with respect to FM. In that study, a notable proportion of general practitioners (36%) and specialists (25%) doubted their ability to diagnose FM. Deficiencies in the treatment of FM were also reported, particularly in the knowledge of current treatment options and in the knowledge of symptom monitoring tools. As with our findings, general practitioners reported deficiencies in diagnosing and treating FM more frequently than specialists.

An important limitation of all opinion research, and this study is not an exception, is that respondents (physicians in this case) may not perfectly recall and assess their experiences. Respondents’ attitudes and perceptions are subject to some potential changes in the course of time. The survey provides a snapshot of the respondents’ experiences and does not seek to address how these might have changed longitudinally. It must also be noted that the questionnaire is limited in that answers are framed in the “yes/no” or 5-point Likert scale formats, which cannot capture detailed accounts of physician experience or additional, unanticipated responses. Finally, these surveys were conducted across multiple countries having different healthcare systems and with physicians coming from different cultural backgrounds. Therefore, care must be taken in attempting to generalize our findings to physicians from countries not included in the current study.

A key finding of our research is that many physicians in the countries surveyed, particularly PCPs, report an overall lack of knowledge and skill in the diagnosis and treatment of FM. Recently, steps have been taken to simplify the diagnosis and care of patients with FM that should be brought to the attention of such physicians. New ACR guidelines, for example, disregard the tender-point examination that was often problematic for physicians lacking a background in rheumatology and employ a severity scale for monitoring common FM symptoms [19]. Newly developed tools, such as the Fibromyalgia Rapid Screening Tool (FiRST) and the VASFIQ Brief Symptom Scale, also simplify symptom assessment. Unlike the FIQ, which can be limited by its length and scoring complexity, the FiRST and the VASFIQ are designed to quickly assess patients and initiate treatment in busy clinics [20,21]. Diagnostic criteria and symptom assessment tools will likely continue to be revised and developed as our understanding of the etiology and care of FM deepens. As such, physicians should try to remain update with the latest guidelines and literature. This should enhance their comfort level with FM and result in improved patient care.

## Conclusions

Fibromyalgia is a challenging disorder to diagnose and manage. Many physicians from a variety of specialties reported difficulties in diagnosing FM, and many were not confident in differentiating the symptoms of FM from other conditions. Physicians and patients commonly report that patients have difficulty communicating symptoms, which may contribute to the diagnostic challenges. The majority of physicians and patients agreed that physicians need to spend more time to identify FM. Over half the physicians surveyed believed that treatments available at the time were inadequate for core symptoms, such as pain and fatigue. Many physicians were not confident in the development of a treatment plan and in the management of FM patients long-term. Many physicians believed their training in FM was inadequate and that they are not knowledgeable about the condition. In general, rheumatologists reported least difficulties/greatest confidence, and PCPs and psychiatrists reported the greatest difficulties/least confidence. Given that meaningful numbers of FM patients are in the care of PCPs and are often also seen by specialists other than rheumatologists, improved training in FM and initiatives to improve patient-doctor communication may help the management of this condition.

## Abbreviations

ACR: American College of Rheumatology; CATI: Computer-assisted telephone interviewing; ENFA: European Network of Fibromyalgia Associations; FM: Fibromyalgia; PCP: Primary care physician; WHO ICD-10: World Health Organization International Statistical Classification of Diseases and Related Health Problems, 10th Revision.

## Competing interests

EC has received fees for consulting to Pfizer. SP has received fees for consulting to Pfizer. AG and EK are employed by Harris Interactive that was commissioned and funded by Pfizer Inc to develop and conduct the survey. The survey was commissioned and funded by Pfizer Inc, New York, NY. Janet Bray, MPharmS, provided medical writing services, which were funded by Pfizer Inc. Journal charges were also met by Pfizer Inc. Editorial support to prepare the manuscript for submission was provided by UBC Scientific Solutions and funded by Pfizer Inc.

## Authors’ contributions

All authors gave final approval of the version submitted for publication. SP and EC contributed to the interpretation of data, were involved in presenting the data and were involved in reviewing and critiquing the manuscript for important intellectual content. DL contributed to the interpretation of data and was involved in reviewing and critiquing the manuscript for important intellectual content. AG made substantial contributions to conception and design, acquisition of data, and analysis and interpretation of data and was involved in reviewing and critiquing the manuscript for important intellectual content. EK made substantial contributions to the conception and design of the survey, interpretation of data and was involved in reviewing and critiquing the manuscript for important intellectual content. All authors read and approved the final manuscript.

## Authors’ information

Profs Perrot and Choy are rheumatologists who have an interest in fibromyalgia and are involved with management of patients and clinical research as well as medical education.

## Pre-publication history

The pre-publication history for this paper can be accessed here:

http://www.biomedcentral.com/1472-6963/12/356/prepub

## Supplementary Material

Additional file 1 Fibromyalgia Global Study - Physician Survey. Click here for file
