# Supplementary material for: Survey of physician experiences and perceptions about the diagnosis and treatment of fibromyalgia
Source: BMC Health Serv Res. 2012 Oct 10;12:356. doi: 10.1186/1472-6963-12-356 (PMC3502453; doi:10.1186/1472-6963-12-356)
Supplement: Additional file 1 — Fibromyalgia Global Study - Physician Survey. [file 1472-6963-12-356-S1.doc]

**HARRIS INTERACTIVE**

**5 Independence Way**

**Princeton, NJ 08543**

**Fibromyalgia Global Study - Physician Survey**

**SECTION 410: SCREENING QUESTIONS**

**BASE: ALL RESPONDENTS**

**Q405** Hello, my name is ______________ from Harris Interactive, an international survey research firm. May I please speak with (INTERVIEWER: INSERT NAME)? (IF NECESSARY: Let me assure you that this is not a sales call, nor will it lead to one, and your answers will be kept strictly confidential.)

1. Yes, speaking ASK Q407
2. Yes, will connect ASK Q407
3. Person unavailable SCHEDULE CALLBACK
4. Person no longer there TERMINATE (NO LONGER THERE)
5. Wrong number TERMINATE (WRONG NUMBER)
6. Not sure (V) TERMINATE (REFUSAL)
7. Decline to answer (V) TERMINATE (REFUSAL)

**BASE: CONTINUING PHONE RESPONDENTS (Q405/1, 2)**

**Q407** (IF NECESSARY: Hello, I’m _______________ with Harris Interactive.) We are conducting a research study about pain and would like to include your opinions. If you qualify for and complete this survey, you will be eligible for an honorarium. Would you be willing to participate in this important research study?

**(INTERVIEWER, IF NECESSARY:** Your responses will be kept strictly confidential and reported in aggregate along with those of other physicians participating in this study.

1. Yes, willing to participate ASK Q410
2. No, not willing to participate TERMINATE (REFUSAL)
3. Willing to participate at a later time SCHEDULE CALLBACK
4. Not sure (V) TERMINATE (REFUSAL)
5. Decline to answer (V) TERMINATE (REFUSAL)

**BASE: CONTINUING PHONE RESPONDENTS (Q407/1)**

**Q410** First we would like to ask a few general questions.

What is your primary medical specialty?

(INTERVIEWER: Do not read list. SINGLE RESPONSE.)

1. General/Family Practitioner [DO NOT DISPLAY FOR Q455/8]
2. Internist
3. Rheumatologist
4. Neurologist
5. Psychiatrist
6. Pain Specialist SKIP TO Q480
7. Anesthesiologist ONLY DISPLAY IF Q455/7

96 Other TERMINATE AND SKIP TO Q480

98 Not sure (V) TERMINATE AND SKIP TO Q480

99 Decline to answer (V) TERMINATE AND SKIP TO Q480

**IF Q410/1-5, 7 CONTINUE WITH Q415, ALL OTHERS SKIP TO Q480.**

**BASE: RESPONDENTS FROM ONE OF THE LISTED SPECIALTIES EXCEPT PAIN SPECIALISTS (Q410/1-5,7)**

**Q415** Do you have a sub-specialty in pain treatment or pain management?

1. Yes

2. No

8. Not sure (V)

9. Decline to answer (V)

**BASE: ALL RESPONDENTS**

**Q480 –** INITIAL CLASSIFICATION QUESTION – BEHIND THE SCENES

PROGRAMMER NOTE:

GET CODE 1 IF:

- Willing to continue (Q405/1,2) AND
- Willing to participate (Q407/1) AND
- Primary specialty is General/Family Practitioner, Internist, Rheumatologist, Neurologist, Psychiatrist, Pain Specialist or Anesthesiologist (Q410/1-7) AND
- Reside in UK, France, Germany, Spain, Italy, Netherlands, Mexico or S. Korea (Q455/1-8)

ALL OTHERS GET CODE 2

1. Qualified
2. Not Qualified

**BASE: ALL QUALIFIED RESPONDENTS (Q480/1)**

**Q485** – COUNTRY/SPECIALTY QUOTA QUESTION – BEHIND THE SCENES

[PN: MULTIPLE RESPONSE]

**UNITED KINGDOM**

GET CODE 1 IF Q455/1 AND Q410/1

GET CODE 2 IF Q455/1 AND Q410/3

GET CODE 3 IF Q455/1 AND Q410/4

GET CODE 4 IF Q455/1 AND Q410/5

GET CODE 5 IF Q455/1 AND (Q410/6 OR (Q410/1-5 AND Q415/1)

**FRANCE**

GET CODE 11 IF Q455/2 AND Q410/1

GET CODE 12 IF Q455/2 AND Q410/3

GET CODE 13 IF Q455/2 AND Q410/4

GET CODE 14 IF Q455/2 AND Q410/5

GET CODE 15 IF Q455/2 AND Q410/6 OR (Q410/1-5 AND Q415/1)

**GERMANY**

GET CODE 21 IF Q455/3 AND Q410/1

GET CODE 22 IF Q455/3 AND Q410/3

GET CODE 23 IF Q455/3 AND Q410/4

GET CODE 24 IF Q455/3 AND Q410/5

GET CODE 25 IF Q455/3 AND (Q410/6 OR (Q410/1-5 AND Q415/1)

**ITALY**

GET CODE 31 IF Q455/4 AND Q410/1

GET CODE 32 IF Q455/4 AND Q410/3

GET CODE 33 IF Q455/4 AND Q410/4

GET CODE 34 IF Q455/4 AND Q410/5

GET CODE 35 IF Q455/4 AND (Q410/6 OR (Q410/1-5 AND Q415/1)

**SPAIN**

GET CODE 41 IF Q455/5 AND Q410/1

GET CODE 42 IF Q455/5 AND Q410/3

GET CODE 43 IF Q455/5 AND Q410/4

GET CODE 44 IF Q455/5 AND Q410/5

GET CODE 45 IF Q455/5 AND (Q410/6 OR (Q410/1-5 AND Q415/1)

**NETHERLANDS**

GET CODE 51 IF Q455/6 AND Q410/1

GET CODE 52 IF Q455/6 AND Q410/3

GET CODE 53 IF Q455/6 AND Q410/4

GET CODE 54 IF Q455/6 AND Q410/5

GET CODE 55 IF Q455/6 AND (Q410/6 OR (Q410/1-5 AND Q415/1)

**MEXICO**

GET CODE 61 IF Q455/7 AND Q410/1

GET CODE 62 IF Q455/7 AND Q410/3

GET CODE 63 IF Q455/7 AND Q410/4

GET CODE 64 IF Q455/7 AND Q410/5

GET CODE 65 IF Q455/7 AND (Q410/6 OR (Q410/1-5, 7 AND Q415/1)

**KOREA**

GET CODE 71 IF Q455/8 AND Q410/2

GET CODE 72 IF Q455/8 AND Q410/3

GET CODE 73 IF Q455/8 AND Q410/4

GET CODE 74 IF Q455/8 AND Q410/5

GET CODE 75 IF Q455/8 AND (Q410/6 OR (Q410/2-5 AND Q415/1)

1. UK PCP N=100
2. UK RHEUMATOLOGIST N=25
3. UK NEUROLOGIST N=25
4. UK PSYCHIATRIST N=25
5. UK PAIN SPECIALIST N=25
6. France PCP N=100
7. France RHEUMATOLOGIST N=25
8. France NEUROLOGIST N=25
9. France PSYCHIATRIST N=25
10. France PAIN SPECIALIST N=25
11. Germany PCP N=100
12. Germany RHEUMATOLOGIST N=25
13. Germany NEUROLOGIST N=25
14. Germany PSYCHIATRIST N=25
15. Germany PAIN SPECIALIST N=25
16. Italy PCP N=100
17. Italy RHEUMATOLOGIST N=25
18. Italy NEUROLOGIST N=25
19. Italy PSYCHIATRIST N=25
20. Italy PAIN SPECIALIST N=25
21. Spain PCP N=100
22. Spain RHEUMATOLOGIST N=25
23. Spain NEUROLOGIST N=25
24. Spain PSYCHIATRIST N=25
25. Spain PAIN SPECIALIST N=25
26. Netherlands PCP N=100
27. Netherlands RHEUMATOLOGIST N=25
28. Netherlands NEUROLOGIST N=25
29. Netherlands PSYCHIATRIST N=25
30. Netherlands PAIN SPECIALIST N=25
31. Mexico PCP N=100
32. Mexico RHEUMATOLOGIST N=25
33. Mexico NEUROLOGIST N=25
34. Mexico PSYCHIATRIST N=25
35. Mexico PAIN SPECIALIST N=25
36. Korea INTERNIST N=100
37. Korea RHEUMATOLOGIST N=25
38. Korea NEUROLOGIST N=25
39. Korea PSYCHIATRIST N=25
40. Korea PAIN SPECIALIST N=25

99. NONE [ALL OTHERS]

**BASE: ALL QUALIFIED RESPONDENTS (Q485/1-75)**

**Q490** – COUNTRY/SPECIALTY- QUOTA TO GO ASSIGNMENT

[PN: GET CODES SELECTED FROM Q485/1-75 FOR QUOTA TO GO SET-UP]

[PN: IF Q410/1-5, 7 AND Q415/1, ASSIGN RESPONDENTS TO THE QUOTA WITH THE LOWEST NUMBER OF COMPLETES. IF ANY QUOTA IS FULL, ASSIGN RESPONDENTS TO THE NEXT OPEN QUOTA.]

**UNITED KINGDOM**

GET CODE 1 IF Q455/1 AND Q410/1

GET CODE 2 IF Q455/1 AND Q410/3

GET CODE 3 IF Q455/1 AND Q410/4

GET CODE 4 IF Q455/1 AND Q410/5

GET CODE 5 IF Q455/1 AND (Q410/6 OR (Q410/1-5 AND Q415/1)

**FRANCE**

GET CODE 11 IF Q455/2 AND Q410/1

GET CODE 12 IF Q455/2 AND Q410/3

GET CODE 13 IF Q455/2 AND Q410/4

GET CODE 14 IF Q455/2 AND Q410/5

GET CODE 15 IF Q455/2 AND Q410/6 OR (Q410/1-5 AND Q415/1)

**GERMANY**

GET CODE 21 IF Q455/3 AND Q410/1

GET CODE 22 IF Q455/3 AND Q410/3

GET CODE 23 IF Q455/3 AND Q410/4

GET CODE 24 IF Q455/3 AND Q410/5

GET CODE 25 IF Q455/3 AND (Q410/6 OR (Q410/1-5 AND Q415/1)

**ITALY**

GET CODE 31 IF Q455/4 AND Q410/1

GET CODE 32 IF Q455/4 AND Q410/3

GET CODE 33 IF Q455/4 AND Q410/4

GET CODE 34 IF Q455/4 AND Q410/5

GET CODE 35 IF Q455/4 AND (Q410/6 OR (Q410/1-5 AND Q415/1)

**SPAIN**

GET CODE 41 IF Q455/5 AND Q410/1

GET CODE 42 IF Q455/5 AND Q410/3

GET CODE 43 IF Q455/5 AND Q410/4

GET CODE 44 IF Q455/5 AND Q410/5

GET CODE 45 IF Q455/5 AND (Q410/6 OR (Q410/1-5 AND Q415/1)

**NETHERLANDS**

GET CODE 51 IF Q455/6 AND Q410/1

GET CODE 52 IF Q455/6 AND Q410/3

GET CODE 53 IF Q455/6 AND Q410/4

GET CODE 54 IF Q455/6 AND Q410/5

GET CODE 55 IF Q455/6 AND (Q410/6 OR (Q410/1-5 AND Q415/1)

**MEXICO**

GET CODE 61 IF Q455/7 AND Q410/1

GET CODE 62 IF Q455/7 AND Q410/3

GET CODE 63 IF Q455/7 AND Q410/4

GET CODE 64 IF Q455/7 AND Q410/5

GET CODE 65 IF Q455/7 AND (Q410/6 OR (Q410/1-5, 7 AND Q415/1)

**KOREA**

GET CODE 71 IF Q455/8 AND Q410/2

GET CODE 72 IF Q455/8 AND Q410/3

GET CODE 73 IF Q455/8 AND Q410/4

GET CODE 74 IF Q455/8 AND Q410/5

GET CODE 75 IF Q455/8 AND (Q410/6 OR (Q410/2-5 AND Q415/1)

1. UK PCP N=100
2. UK RHEUMATOLOGIST N=25
3. UK NEUROLOGIST N=25
4. UK PSYCHIATRIST N=25
5. UK PAIN SPECIALIST N=25

11. France PCP N=100

12. France RHEUMATOLOGIST N=25

1. France NEUROLOGIST N=25
2. France PSYCHIATRIST N=25
3. France PAIN SPECIALIST N=25

21. Germany PCP N=100

22. Germany RHEUMATOLOGIST N=25

- 1. Germany NEUROLOGIST N=25
  2. Germany PSYCHIATRIST N=25
  3. Germany PAIN SPECIALIST N=25
  4. Italy PCP N=100
  5. Italy RHEUMATOLOGIST N=25
  6. Italy NEUROLOGIST N=25
  7. Italy PSYCHIATRIST N=25
  8. Italy PAIN SPECIALIST N=25
  9. Spain PCP N=100
  10. Spain RHEUMATOLOGIST N=25
  11. Spain NEUROLOGIST N=25
  12. Spain PSYCHIATRIST N=25
  13. Spain PAIN SPECIALIST N=25
  14. Netherlands PCP N=100
  15. Netherlands RHEUMATOLOGIST N=25
  16. Netherlands NEUROLOGIST N=25

54. Netherlands PSYCHIATRIST N=25

55. Netherlands PAIN SPECIALIST N=25

- 1. Mexico PCP N=100
  2. Mexico RHEUMATOLOGIST N=25
  3. Mexico NEUROLOGIST N=25
  4. Mexico PSYCHIATRIST N=25
  5. Mexico PAIN SPECIALIST N=25

1. Korea PCP (INTERNIST) N=100
2. Korea RHEUMATOLOGIST N=25
3. Korea NEUROLOGIST N=25
4. Korea PSYCHIATRIST N=25
5. Korea PAIN SPECIALIST N=25

97. ALL QUOTAS MET

**BASE: ALL RESPONDENTS**

**Q486** COUNTRY/SPECIALTY QUOTA CHECK QUESTION

[PROGRAMMER NOTE: CHECK QUOTA AT Q490.]

1 QUOTA MET

2 QUOTA NOT MET

3 QUOTA NOT FOUND

**BASE: ALL RESPONDENTS**

**Q99** SCREENER QUALIFICATION IDENTIFICATION QUESTION (DOES NOT APPEAR ON SCREEN)

[PN: GET CODE 1 IF Q480/1 AND Q490/1-80 AND Q486/2. GET CODE 3 IF Q480/1 AND Q490/1-80 AND Q486/1. ALL OTHERS GET CODE 6.

1 SCREENER QUALIFIED RESPONDENTS, QUOTA OPEN

3 SCREENER QUALIFIED RESPONDENTS, QUOTA CLOSED

6 NOT SCREENER QUALIFIED

**SECTION 400: EXPERIENCE/KNOWLEDGE OF FIBROMYALGIA**

**BASE: ALL QUALIFIED RESPONDENTS (Q99/1)**

**Q435** For how many years have you been in practice?

(INTERVIEWER, ENTER ‘98’ FOR NOT SURE OR ‘99’ FOR DECLINE TO ANSWER)

[RANGE: 0-65, 98, 99]

|__|__| year(s)

**BASE: ALL QUALIFIED RESPONDENTS (Q99/1)**

**Q440** Approximately how many patients per month do you see yourself in your practice overall? *Your best estimate is fine.*

(INTERVIEWER: Enter 9998 for Not Sure, and 9999 for Decline to Answer.)

[RANGE 0-9999]

|_|_|_|_

**BASE: ALL QUALIFIED RESPONDENTS (Q99/1)**

**Q445** Are you currently seeing or have you seen patients over the past 2 years diagnosed with the following conditions?

(INTERVIEWER: Read list. Allow multiple responses.)

[RANDOMIZE]

1. Migraines

2. Arthritis

3. Chronic fatigue syndrome

4. Depression

5. Irritable bowel syndrome

6. Lyme disease

7. Fibromyalgia

8.. Lupus

9. Multiple Sclerosis

97. None of the above [ANCHOR, E]

98. Not sure (ANCHOR, V, E)

99. Decline to answer (ANCHOR, V, E)

**IF Q445/7 CONTINUE WITH Q460. IF Q445/NE7, SKIP TO Q470.**

**BASE: CURRENTLY SEEING/HAVE SEEN PATIENTS DIAGNOSED WITH FIBROMYALGIA (Q445/7)**

**Q460** Approximately, how many patients diagnosed with fibromyalgia have you seen in the past 2 years including those you are currently seeing?

(INTERVIEWER: Enter ‘998’ for Not Sure, ‘999’ for Decline to Answer.)

[RANGE 1-999]

|_|_|_|

**BASE: CURRENTLY SEEING/HAVE SEEN PATIENTS DIAGNOSED WITH FIBROMYALGIA (Q445/7)**

**Q465** What percentage of the fibromyalgia patients that you have seen in the past 2 years, including those you are currently seeing, are women?

(INTERVIEWER: Enter ‘998’ for Not Sure, ‘999’ for Decline to Answer.)

[RANGE 0-100, 998, 999]

|_|_|_| % women

**BASE: ALL QUALIFIED RESPONDENTS (Q99/1)**

**Q470** Now, I would like to ask you some general questions about fibromyalgia. How would you rate your level of knowledge about fibromyalgia? Would you say you are…?

1. Very knowledgeable
2. Knowledgeable
3. Not very knowledgeable
4. Not at all knowledgeable

8. Not sure (V)

9. Decline to answer (V)

**IF Q445/7 CONTINUE WITH Q500. All OTHERS SKIP TO Q515.**

**SECTION 500: DIAGNOSIS OF FIBROMYALGIA**

**BASE: CURRENTLY SEEING/SEEN PATIENTS WITH FIBROMYALGIA (Q445/7)**

**Q500** Now, please think of all the patients you have seen with fibromyalgia in the past 2 years, including those you are currently seeing. Which of the following statements describe the ways you handle diagnosis and treatment of your fibromyalgia patients? *(INTERVIEWER: READ EACH ITEM. MARK ALL THAT APPLY)*?

[MULTIPLE RESPONSE]

1. You diagnose and then refer your fibromyalgia patients to another physician for treatment

2. You diagnose and also treat your fibromyalgia patients

3. You treat fibromyalgia patients who were diagnosed by another physician and then referred to you for treatment

8. Not sure (V)

9. Decline to answer (V)

**IF Q500/3 CONTINUE WITH Q505. IF Q500/1 SKIP TO Q510. IF Q500/2 SKIP TO Q515.**

**BASE: HAVE PATIENTS WHO WERE REFERRED TO THEM (Q500/3)**

**Q505** Thinking about the patients who were diagnosed by another physician and then referred to you for treatment over the past 2 years, which physician specialties commonly refer fibromyalgia patients to you? *(INTERVIEWER: READ LIST. ALLOW MULTIPLE RESPONSES. IF RESPONDENT MENTIONS A SPECIALTY NOT LISTED, CODE UNDER ‘OTHER*’’.*)*

1. General / Family Practitioner
2. Internists
3. Rheumatologists
4. Neurologists
5. Psychiatrists
6. Pain Specialists
7. OBGYN
8. Orthopedists
9. Anesthesiologists
10. Physical Therapists

96 Other

98 Not sure (V, E)

99 Decline to answer (V, E)

**BASE: HAVE PATIENTS WHO HAVE DIAGNOSED AND THEN REFERRED (Q500/1)**

**Q510** You have indicated that over the past 2 years you have referred fibromyalgia patients to another physician for treatment. Why did you refer those patients to another physician? Would you say…?

*(INTERVIEWER: READ LIST. MARK ALL THAT APPLY.)*

[MULTIPLE RESPONSE]

1. You were not familiar with treatments appropriate for patients with fibromyalgia
2. You had limited experience with fibromyalgia

3.   You did not have time to treat fibromyalgia patients over the long term

8. Not sure (V, E)

9.   Decline to answer (V, E)

**BASE: ALL QUALIFIED RESPONDENTS (Q99/1)**

**Q515** From your experience and/or what you may have read or heard, how easy or how difficult is it to diagnose fibromyalgia? Would you say…? *(INTERVIEWER: READ LIST)*

1. Very easy

2. Somewhat easy

3. Neither easy nor difficult

4. Somewhat difficult

5. Very difficult

8. Not sure (V)

9. Decline to answer (V)

**BASE: ALL QUALIFIED RESPONDENTS (Q99/1)**

**Q520** I am going to read you several statements some physicians make about diagnosing fibromyalgia. From your experience and/or what you may have read or heard, please tell me if you strongly agree , somewhat agree , neither agree nor disagree, somewhat disagree, or strongly disagree with each of the following statements.

**Q521**

Strongly Somewhat Neither Agree Somewhat Strongly Not Decline to

Agree Agree Nor Disagree Disagree Disagree Sure (V) Answer (V)

1 2 3 4 5 8 9

*(INTERVIEWER: READ LIST. REPEAT SCALE AS NECESSARY.)*

[RANDOMIZE]

1. It is difficult for patients to communicate symptoms of fibromyalgia to a physician

2. Patients tend to delay seeking medical help even when symptoms of fibromyalgia appear

3. Physicians are not always comfortable diagnosing fibromyalgia

4. The symptoms of fibromyalgia can often be difficult to discriminate from the symptoms of other conditions

5. Physicians need to spend more time with patients to identify fibromyalgia

**BASE: ALL QUALIFIED RESPONDENTS (Q99/1)**

**Q525** From your experience and/or what you may have read or heard, how often do you think fibromyalgia is misdiagnosed? Would you say…?

1. Almost always

2. Often

3. Sometimes

4. Rarely

5. Never

8. Not sure (V)

9. Decline to answer (V)

**BASE: ALL QUALIFIED RESPONDENTS (Q99/1)**

**Q530**  And how confident are you personally about each of the following? Would you say you are very confident, confident, not very confident, or not at all confident?

Q531

Very Confident Not Very Not At All Not Decline to

Confident Confident Confident Sure (V) Answer (V)

1 2 3 4 8 9

*(INTERVIEWER: READ LIST. REPEAT SCALE AS NECESSARY.)*

**[DO NOT RANDOMIZE]**

1. Recognizing the symptoms of fibromyalgia

2. Differentiating fibromyalgia from conditions with similar symptoms

3. Conducting tender points examination

4. Developing treatment plans for patients diagnosed with fibromyalgia

5. Managing fibromyalgia patients over the long-term

**BASE: ALL QUALIFIED RESPONDENTS (Q99/1)**

**Q535** Which of the following best describes the level of fibromyalgia training that you received? *(INTERVIEWER: READ LIST. SINGLE RESPONSE)*

- 1. You received excellent fibromyalgia training
  2. You received adequate fibromyalgia training
  3. You received very little fibromyalgia training
  4. You did not receive any fibromyalgia training at all

8 Not sure (V)

9 Decline to answer (V)

**BASE: ALL QUALIFIED RESPONDENTS (Q99/1)**

**Q540** Are you aware of the fibromyalgia diagnostic criteria published by the American College of Rheumatology (ACR) in 1990?

1. Yes

2. No

8. Not sure (V)

9. Decline to answer (V)

**IF Q540/1 AND Q445/7 CONTINUE WITH Q545. All OTHERS SKIP TO Q600.**

**BASE: CURRENTLY SEEING/HAVE SEEN PATIENTS WITH FIBROMYALGIA AND ARE AWARE OF CRITERIA (Q445/7 AND Q540/1)**

**Q545** Do you use the American College of Rheumatology (ACR) diagnostic criteria to identify fibromyalgia in your clinical practice?

1. Yes

2. No

8. Not sure (V)

9. Decline to answer (v)

**IF Q545/2, 8. 9 CONTINUE WITH Q550. All OTHERS SKIP TO Q600.**

**BASE: DO NOT USE CRITERIA IN PRACTICE (Q545/2, 8, 9)**

**Q550** I’m going to read you several statements that describe the reasons why you may not use the ACR criteria to identify fibromyalgia. For each statement, please tell me if it is a reason why you would not use the ACR criteria. [*INTERVIEWER: READ EACH STATEMENT FROM THE LIST AND AFTER EACH STATEMENT SAY: ‘*Is it a reason why you would not use the ACR criteria*?’]*

Q551

Yes No Not Sure (V) Decline to Answer (V)

1 2 8 9

[RANDOMIZE]

1. You do not feel that the usage of the criteria yields an accurate diagnosis
2. You do not have enough time to use ACR criteria
3. You depend more on your own clinical judgment than diagnostic criteria
4. You do not have sufficient experience using ACR criteria

**IF Q445/NE7 CONTINUE WITH Q600. IF Q445/7 SKIP TO Q605.**

**SECTION 600: SYMPTOMS OF FIBROMYALGIA**

**BASE: CURRENTLY NOT SEEING / HAVE NOT SEEN PATIENTS WITH FIBROMYALGIA (Q445/NE7)**

**Q600** Next, I am going to read you some symptoms of fibromyalgia. Based on what you may have read or heard about fibromyalgia, please tell me how typical each one is as a symptom of the disease – most typical, very typical, typical, not too typical, or not at all typical. (*INTERVIEWER: READ LIST AND REPEAT SCALE IF NECESSARY.)*

Q601

Most Very Typical Not Too Not At All Not Decline to

Typical Typical Typical Typical Sure(V) Answer (V)

1 2 3 4 5 8 9

[RAMDOMIZE]

1. Chronic widespread pain

2. Problems sleeping

3. Fatigue

4. Headaches

5. Facial pain

6. Heightened sensitivity to touch

7. Difficulty concentrating

8. Numbness and/or tingling sensations

9. Feelings of anxiety

10. Feelings of depression

11. Joint pain

12. Stiffness

13. Leg cramps

14. Low back pain

**IF Q445/NE7 SKIP TO Q700. IF Q445/7 ASK Q605.**

**BASE: CURRENTLY SEEING / HAVE SEEN PATIENTS WITH FIBROMYALGIA (Q445/7)**

**Q605** Next, I am going to read you a list of symptoms of fibromyalgia. What is the one most typical symptom you look for when diagnosing this condition? *(INTERVIEWER: READ LIST. SINGLE RESPONSE.)*

[RAMDOMIZE]

1. Chronic widespread pain

2. Problems sleeping

3. Fatigue

4. Headaches

5. Facial pain

6. Heightened sensitivity to touch

7. Difficulty concentrating

8. Numbness and/or tingling sensations

9. Feelings of anxiety

10. Feelings of depression

11. Joint pain

12. Stiffness

13. Leg cramps

14. Low back pain

97. None of the above (V, E) [ANCHOR]

98. Not sure (V, E) [ANCHOR]

99. Decline to answer (V, E) [ANCHOR

**BASE: CURRENTLY SEEING / HAVE SEEN PATIENTS WITH FIBROMYALGIA (Q445/7)**

**Q610** Again, thinking about the symptoms of fibromyalgia, how disruptive you think each of them is to the overall quality of the patients’ lives? Would you say it is not at all disruptive, not very disruptive, fairly disruptive, very disruptive, or extremely disruptive? (*INTERVIEWER: READ LIST AND REPEAT SCALE IF NECESSARY)*

Q611

Not At All Not very Fairly Very Extremely Not Decline to

Disruptive Disruptive Disruptive Disruptive Disruptive Sure (V) Answer (V)

1 2 3 4 5 8 9

[RANDOMIZE]

1. Chronic widespread pain
2. Problems sleeping
3. Fatigue
4. Headaches
5. Facial pain
6. Heightened sensitivity to touch
7. Difficulty concentrating
8. Numbness and/or tingling sensations
9. Feelings of anxiety
10. Feelings of depression
11. Joint pain
12. Stiffness
13. Leg cramps
14. Low back pain

**SECTION 700: ATTITUDES AND BELIEFS ABOUT FIBROMYALGIA**

**BASE: ALL QUALIFIED RESPONDENTS (Q99/1)**

**Q700** Now, I will read you several general statements about fibromyalgia. Based on your experience and/or what you may have read or heard, please indicate whether you strongly agree, somewhat agree, neither agree nor disagree, somewhat disagree, or strongly disagree with each of the following statements? (*INTERVIEWER READ LIST. REPEAT SCALE AS NECESSARY)*

Q701

Strongly Somewhat Neither Agree Somewhat Strongly Not Decline to

Agree Agree Nor Disagree Disagree Disagree Sure (V) Answer (V)

1 2 3 4 5 8 9

[RANDOMIZE]

1. Physicians are well trained to diagnose and treat fibromyalgia
2. Physicians are compassionate with their fibromyalgia patients
3. Most fibromyalgia patients have had at least one experience of a physician not taking them seriously
4. Fibromyalgia patients exaggerate their symptoms
5. Physicians need to focus more on fibromyalgia symptoms

**IFQ445/7 CONTINUE WITH Q705. ALL OTHERS SKIP TO Q805.**

**BASE: CURRENTLY SEEING / HAVE SEEN PATIENTS WITH FIBROMYALGIA (Q445/7)**

**Q705** And now I would like to ask you about the impact of fibromyalgia on your patients’ lives. I am going to read you a list of some aspects of your patients’ lives that may have been impacted by fibromyalgia. Please tell me if you think fibromyalgia has had a very strong impact, strong impact, moderate impact, slight impact or no impact on each of the following aspects.

(INTERVIEWER: READ OUT LIST AND REPEAT SCALE IF NECESSARY.)

Q706

Very Strong Strong Moderate Slight No Not Decline to

Impact Impact Impact Impact Impact Sure (V) Answer (V)

1 2 3 4 5 8 9

[RANDOMIZE]

1. Overall quality of patients lives [ANCHOR]
2. Their personal relationships
3. Their ability to keep commitments or appointments
4. Their ability to participate in hobbies
5. Their ability to care for family members and children
6. Their sex life
7. Their physical mobility
8. Their overall mood
9. Their concentration or memory
10. Their motivation or drive
11. Their ability to work
12. Their finances

**SECTION 800: TREATING FIBROMYALGIA**

**BASE: ALL QUALIFIED RESPONDENTS (Q99/1)**

**Q805** Now, I am going to read you several statements about treating fibromyalgia. From your experience and/or what you may have read or heard about treating fibromyalgia, please indicate whether you strongly agree, somewhat agree, neither agree nor disagree, somewhat disagree, or strongly disagree with each of the following statements? (*INTERVIEWER READ LIST. REPEAT SCALE AS NECESSARY.)*

Q806

Strongly Somewhat Neither Agree Somewhat Strongly Not Decline to

Agree Agree Nor Disagree Disagree Disagree Sure (V) Answer (V)

1 2 3 4 5 8 9

[RANDOMIZE]

1. Treating fibromyalgia can be very time consuming for physicians

2. Treating fibromyalgia requires frequent patient visits

3. It is difficult to gauge progress in treating fibromyalgia

4. With so many symptoms of fibromyalgia, many different medications are needed to treat it

**IF Q445/7 CONTINUE. ALL OTHERS SKIP TO Q825.**

**BASE: CURRENTLY SEEING OR HAVE SEEN FIBROMYALGIA PATIENTS (Q445/7)**

**Q810** Overall, how would you rate the currently available treatments for fibromyalgia? Would you say…? *(INTERVIEWER: READ LIST)*

1. Excellent

2. Very good

1. Good
2. Fair

5. Poor

8. Not sure (V)

9. Decline to answer (V)

**BASE: CURRENTLY SEEING OR HAVE SEEN FIBROMYALGIA PATIENTS (Q445/7)**

**Q820** Overall, what symptom(s) of fibromyalgia, if any, do you feel are not adequately treated by current treatment options? (*INTERVIEWER: READ LIST. ALLOW MULTIPLE RESPONSES.)*

[RANDOMIZE]

1. Chronic widespread pain
2. Problems sleeping
3. Fatigue
4. Headaches
5. Facial pain
6. Heightened sensitivity to touch
7. Difficulty concentrating
8. Numbness and/or tingling sensations
9. Feelings of anxiety
10. Feelings of depression
11. Joint pain
12. Stiffness
13. Leg cramps
14. Low back pain

97. None of the above (V, E) [ANCHOR]

98. Not sure (V, E) [ANCHOR]

99. Decline to answer (V, E) [ANCHOR]

**BASE: ALL QUALIFIED RESPONDENTS (Q99/1)**

**Q825** Do you have specific fibromyalgia treatment guidelines in your country?

1. Yes

2. No

8. Not sure (V)

9. Decline to answer (V)

**IF Q825/1 AND Q445/7 CONTINUE WITH Q830. All OTHERS SKIP TO Q835.**

**BASE: CURRENTLY SEEING OR HAVE SEEN FIBROMYALGIA PATIENTS AND HAVE SPECIFIC TREATMENT GUIDELINES (Q445/7 AND Q825/1)**

**Q830** Do you use the fibromyalgia treatment guidelines in your practice?

1. Yes

2. No

8. Not sure (V)

9. Decline to answer (V)

**BASE: ALL QUALIFIED RESPONDENTS (Q99/1)**

**Q835** To your knowledge, are there any medications specifically approved for the treatment of fibromyalgia in your country?

1. Yes

2. No

8. Not sure (V)

9. Decline to answer (V)

**SECTION 900: INCLUSION OF FIBROMYALGIA INTO INSTITUTION FORMULARY**

**BASE: ALL QUALIFIED RESPONDENTS (Q99/1)**

**Q900** To the best of your knowledge, is fibromyalgia coded in the official index of conditions in your country’s healthcare system or institution formulary?

1. Yes

2. No

8. Not sure (V)

9. Decline to answer (V)

**IF Q900/2,8,9 CONTINUE WITH Q905. IF Q900/1 SKIP TO Q910.**

**BASE: RESPONDENTS WHO STATE THAT FIBROMYALGIA IS NOT INCLUDED IN THE LIST (Q900/2,8,9)**

**Q905** How important do you think coding fibromyalgia in the official index of conditions is for improved fibromyalgia patient care? Would you say…? (INTERVIEWER: READ LIST)

1. Absolutely essential

2. Very important

3. Fairly important

4. Not very important

5. Not at all important

8. Not sure (V)

9. Decline to answer (V)

**BASE: ALL QUALIFIED RESPONDENTS (Q99/1)**

**Q910** Now, I’m going to read several statements that describe the benefits of coding fibromyalgia in the official index of conditions used in your country’s healthcare system or institution formulary. Please indicate whether you strongly agree, somewhat agree, neither agree nor disagree, somewhat disagree, or strongly disagree with each of the following statements? (*INTERVIEWER READ LIST. REPEAT SCALE AS NECESSARY.)*

Q911

Strongly Somewhat Neither Agree Somewhat Strongly Not Decline to

Agree Agree Nor Disagree Disagree Disagree Sure (V) Answer (V)

1 2 3 4 5 8 9

[RANDOMIZE]

1. Coding fibromyalgia in the official index of conditions improves access to treatment

2. Coding fibromyalgia in the official index of conditions increases awareness of the disease

3. Coding fibromyalgia in the official index of conditions reduces time to accurately diagnose patients

**IF Q445/7 CONTINUE WITH Q1000. IF Q445/NE 7 SKIP TO Q1005.**

**PHYSICIAN REFERAL**

**BASE: RESPONDENTS WHO ARE SEEING/HAVE SEEN FIBROMYALGIA PATIENTS (Q445/7)**

**Q1000** We are also conducting research with Fibromyalgia patients, and would like to ask for your help in recruiting some of **your patients who have been diagnosed with fibromyalgia.** In appreciation, an honorarium in the amount of (INTERVIEWER: INSERT AMOUNT) will be offered for each patient you recruit who qualifies and completes an interview.

You may inform **your patients who have been diagnosed with fibromyalgia** that we are asking them to participate in a survey to learn about their experiences with diagnosis and treatment of fibromyalgia. The survey will be conducted on an anonymous basis. You may also inform patients that their personal anonymity is guaranteed and that results will only be reported in aggregate.

IF Q455/1-7, SAY:

Please ask qualifying patients to call the following toll-free number:

(INTERVIEWER: INSERT INBOUND TOLL-FREE NUMBER)

Again, we are interested in interviewing patients diagnosed with fibromyalgia.

An interviewer will be waiting to take their call between the hours of (INTERVIEWER: INSERT TIME) and (INTERVIEWER: INSERT TIME) every day of the week from (INTERVIEWER: INSERT DATE) to [INSERT DATE]. Instruct them to provide the following ID number to the interviewer: (INTERVIEWER: INSERT ID NUMBER). This number will identify you as the recruiter, so that we can calculate your total honorarium for the patients you recruit who qualify and complete the survey.

You yourself may feel free to call (INTERVIEWER: INSERT VENDOR TOLL FREE NUMBER) if you have additional questions.

**BASE: ALL RESPONDENTS (Q99/1)**

**Q1005** These are all the questions that we have. Thank you very much for your participation in this important research. We greatly appreciate your time and input. Have a great day.
